# A protonic biotransducer controlling mitochondrial ATP synthesis

**DOI:** 10.1038/s41598-018-28435-5

**Published:** 2018-07-12

**Authors:** Z. Zhang, H. Kashiwagi, S. Kimura, S. Kong, Y. Ohta, T. Miyake

**Affiliations:** 1 Graduate School of Information, Production and Systems, Waseda University, Kitakyushu, Fukuoka 808-0135 Japan; 2 Division of Biotechnology and Life Sciences, Institute of Engineering, Tokyo University of Agriculture and Technology, Koganei, Tokyo 184-8588 Japan

## Abstract

In nature, protons (H^+^) play an important role in biological activities such as in mitochondrial ATP synthesis, which is driven by a H^+^ gradient across the inner membrane, or in the activation of acid sensing ion channels in neuron cells. Bioprotonic devices directly interface with the H^+^ concentration (pH) to facilitate engineered interactions with these biochemical processes. Here we develop a H^+^ biotransducer that changes the pH in a mitochondrial matrix by controlling the flow of H^+^ between a conductive polymer of sulfonated polyaniline and solution. We have successfully modulated the rate of ATP synthesis in mitochondria by altering the solution pH. Our H^+^ biotransducer provides a new way to monitor and modulate pH dependent biological functions at the interface between the electronic devices and biological materials.

## Introduction

Controlling the flow of ions and electrons at the device/biological interface has become an important challenge in broad fields such as bioelectronics^1,2^ and medical biology^3,4^. These devices provide a new way to translate bidirectionally between the ionic language of biology and the electronic language of circuitry.

The current strategy to develop such ion and electron controlling devices is to use new functional materials at the device/biological interface. For example, silicon nanowire and carbon nanotube based transistors use ions to read out biological functions^5,6^. Aluminum nanostraws^7^ and carbon nanotube porins^8^ can selectively deliver molecules such as cations^8^, DNA^7^ and nicotine^9^ into biological materials. Since conductive polymers allow the transport of both ions and electrons, there are many conductive polymer based examples including organic field effect transistors for biosensing^10,11^ and organic ion pumps for locally delivering ions and neurotransmitters into cells^12^ and the brain^13^.

**Figure 1:**
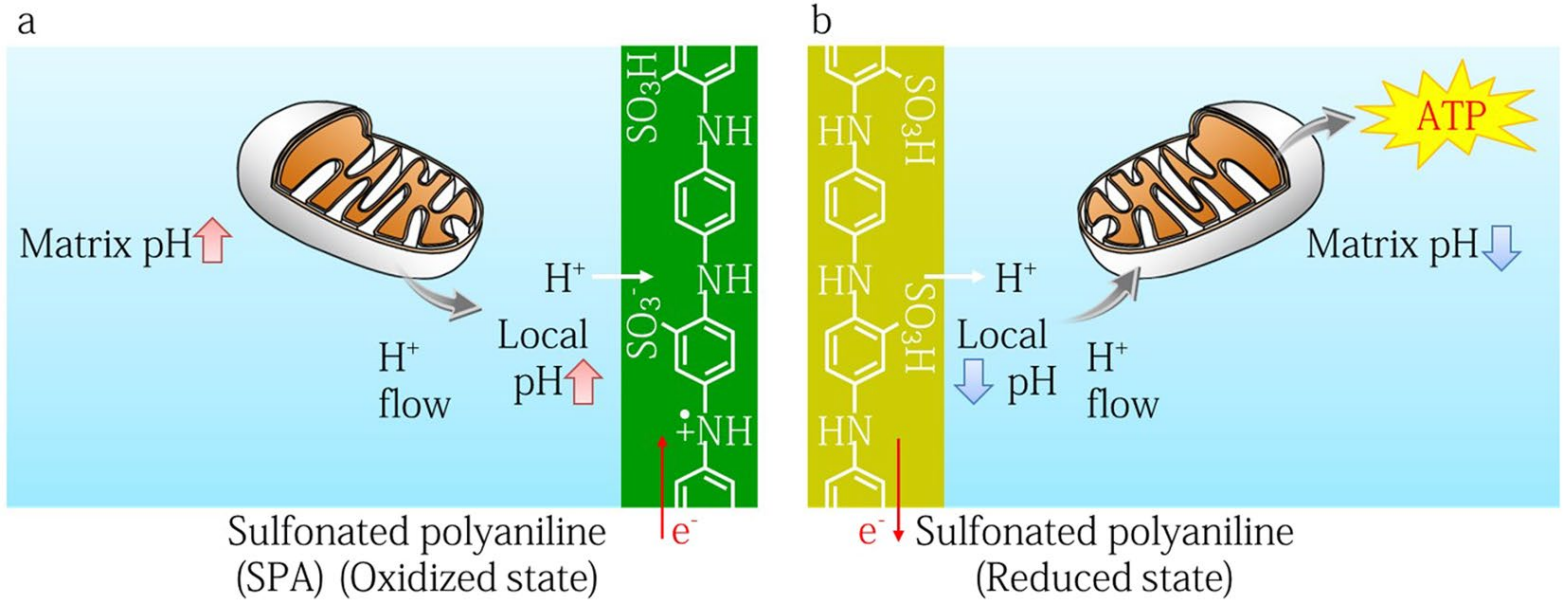
A protonic biotransducer controlling ATP synthesis in mitochondria. Mitochondria synthesize ATP molecules using the proton-motive force, which is generated by a proton (H^+^) concentration gradient and a voltage gradient. Protons are pumped from the exoplasmic face to the cytosolic face, creating a proton concentration gradient (H^+^_exoplasmic face_ > H^+^_cytosolic face_) and a voltage gradient (positive at the cytosolic face and negative at the exoplasmic face) across the membrane. During ATP synthesis, protons flow in the reverse direction through ATP synthase. When the SPA pH modulator transfers H^+^ between the solution and contact, it changes the pH around the SPA microelectrodes. This pH modulation couples to the mitochondrial proton-motive force. (**a**) Proton-motive force (H^+^ concentration in intermembrane space) decreases upon an applied reduced potential to the SPA contact. (**b**) Proton-motive force increases upon an applied oxidizing potential to the SPA contact, resulting in mitochondrial ATP synthesis.

Along with ions and small molecules, protons (H^+^) play an important role in biology^14^. Examples include the homeostatic pH regulation in body^15^ and bacteria^16^, acid sensing ion channels activating in neuron cells^17^, proton activated bioluminescence in dinoflagellates^18^, and pH responsive flagella in bacteria^19^. Mitochondria in particular are a noteworthy organelle that utilize the transport of protons and electrons across a membrane to synthesize adenosine triphosphate (ATP) molecules^20^. To translate H^+^ signals from biological environments into measureable electronic signals, Rolandi and co-workers developed a prototype bioprotonic device using a Pd/PdH_x_ protode^21^. In his group, the metallic protode was used to measure the protonic conductivity in biological materials such as chitosan^21,22^ and jelly from in the ampullae of Lorenzini of sharks^23^, and to integrate electronic signals with an enzymatic flip flop circuit^24^, ion channels^25^, and light-sensitive bacteriorhodopsin^26^. Gorodestky and co-workers have separately demonstrated high proton conductivity in reflectin squid proteins and Pd/PdH_x_ based transistors^27,28^. All attempts however focused on the interfacing between Pd-based metal and biological materials, even though the Pd protode may induce side reactions such as oxygen conversion (PdO, PdO_2_ and Pd(OH)_x_) at the potentials beginning at 0.9 V vs. NHE^29^ and toxic hydrogen peroxide production^30^. In this work, we develop an organic biotransducer using a high H^+^-coupling conductive polymer of sulfonated polyaniline (SPA) that monitors and modulates the pH in the vicinity of the SPA electrode, even in solutions with the high buffering capacity typical of mitochondrial environments (Fig. 1). Among all conductive polymers, polyaniline (PA) and SPA are well-known proton coupling materials that can be used as pH sensors^31^. However, when a voltage (−0.1 to 0.4 V vs. SCE) is applied to a PA electrode in 0.1 M HCl, cationic (H^+^) and anionic (Cl^−^) charges participate in the redox reaction^32^. In contrast, the charge exchange at an SPA electrode is mostly H^+^^33^, so proton flux dominates the redox current. In this work, we confirm the proton affinity of SPA electrodes in an aqueous media by electrochemical measurements and also examine the capabilities of an SPA electrode as compared with common PdH_x_ protode. We integrate this SPA H^+^ biotransducer with mitochondria isolated from pig hearts to translate the H^+^ signal from an activated mitochondria into a measureable protonic current, and also control the activity of ATP synthase through electrochemical pH modulation at the SPA surface.

## Results

### pH regulation in solution with H^+^ transducer

**Figure 2:**
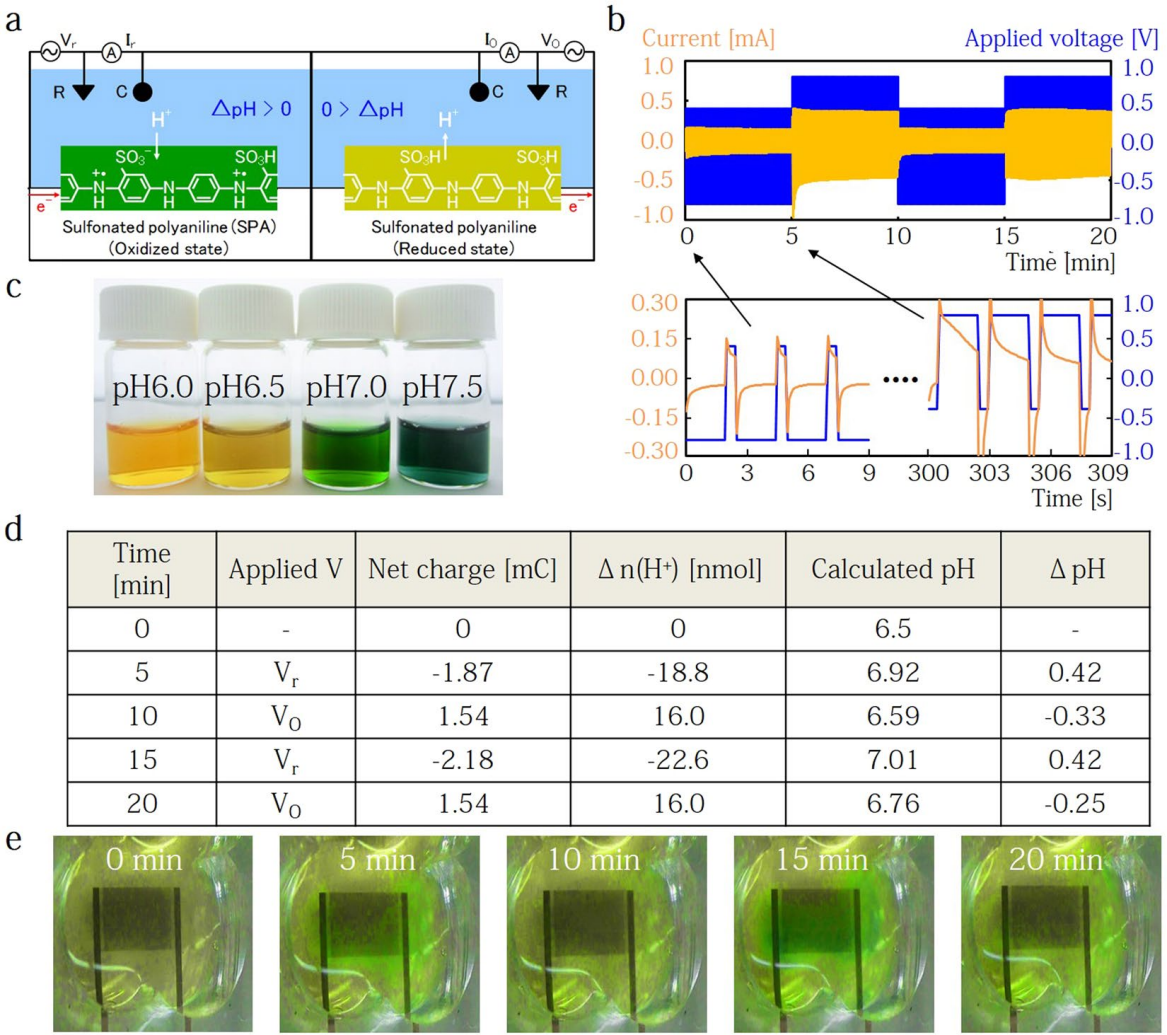
pH regulation in solution. (**a**) Schematics of the pH modulator with sulfonated polyaniline (SPA). In (a), as the potential is decreased (V_r_), the SPA is reduced to neutralize polarons in the polymer chain and transfer H^+^ from the solution to the SO^3−^ groups (H^+^ doping). This decrease of H^+^ concentration causes a pH increase in the solution. In contrast, when the potential is increased to the oxidized SPA, H^+^ move back to the solution (H^+^ dedoping). This transfer returns the pH to the initial acidic state. (**b**) Temporal characteristics of the H^+^ doping into SPA polymer (V_r_ pulse = −0.8 V for 2 s and 0.4 V for 0.5 s) and dedoping (V_o_ = 0.8 V for 2 s and −0.4 V for 0.5 s) in 10 mM Tris-HCl buffer at pH 6.5. (**c**) The bromothymol blue dye at different solution pH. (**d**) A summarized table of net charge Q from the protonic current (I_r_ and I_o_) during H^+^ doping and dedoping. H^+^ change, n, is calculated from Q/F, where F is the faraday constant. The final pH after the applied potential is then estimated from equation (1). (**e**) Pictures of pH modulator with pH dye in solution at t = 0, 5, 10, 15, and 20 min. Yellow tinted dye indicates a solution pH = 6.5, which is the initial pH of solution. Green tinted dye indicate a solution pH = 7.0.

To calibrate the H^+^ transducer, we first investigated the dependence of the acceptance (doping) and donation (dedoping) of protons within the polymer structure on the potential (V) applied to the SPA polymer (Fig. 2). For this measurement, we prepared the electrochemically-polymerized SPA on a Au comb microelectrode (Fig. S1) in a standard three electrode system with Ag/AgCl as the reference electrode and Pt as the counter electrode. We then measured the electrochemical current (I) at the SPA electrode (0.0855 cm^2^ surface area) in 10 mM Tris-HCl buffer solution (pH 6.5). In pH 6.5 solution, negatively charged sulfonate groups that are covalently attached to the SPA backbone act as dopant anions, which are able to compensate positive charges at protonated nitrogen atoms on the SPA backbone. To form the SPA in an oxidized state, we dope protons into the SPA with a reduction V applied to the SPA contact, which we refer to as V_r_. This V_r_ induces an e^−^ reduction current flow (I_r_) into the SPA microelectrodes. These e^−^ neutralize the charge at nitrogen atoms on the SPA backbone, allowing the negatively charged sulfonate groups to couple with protons from the solution (Fig. 2a). In the same manner as proton doping, an oxidation voltage (V_o_) applied to the SPA induces an oxidation current (I_o_), resulting in proton injection from the SPA into the solution. Our results confirm that the redox current peaks are from H^+^ doping and dedoping at the SPA electrode in the different pH solution (Fig. S2). When we decrease the pH from 6.5 to 1.0, the redox peaks shift to positive potentials, and the redox currents increase due to the higher electrochemical potential in solution. This is consistent with the PdH_x_ protode in our previous reports^34^ and others^35^. The pH on the SPA surface (pH_surface_) is defined as^36^.

1 $$p{H}_{surface}={\rm{p}}{K}_{a}+\,\mathrm{log}(\frac{[Tris]-\frac{n({H}^{+})}{V}}{[Tris{H}^{+}]+\frac{n({H}^{+})}{V}})$$

where the ${\rm{p}}{K}_{a}$ of Tris-HCl is 8.1 at 25 °C, [Tris] and [TrisH^+^] are the concentration of 0.268 mM and 9.732 mM, respectively, V is the solution volume, and $n({H}^{+})$ is the total quantity of protons. $n({H}^{+})$ is derived from the equation ${\rm{n}}({H}^{+})=\,{\sum }^{}It/F$, where I is the current, t is the time, and F is Faraday constant.

According to equation (1), H^+^ transfer results in a measurable current and local pH change at the SPA surface. To confirm H^+^ transfer from the SPA electrode into the buffer solution, we applied a pulse potential for H^+^ doping (V_r_ pulse = −0.8 V for 2 s and 0.4 V for 0.5 s, Cycles: 120 (total: 5 min)) and for H^+^ dedoping (V_o_ pulse: 0.8 V for 2 s and −0.4 V for 0.5 s, Cycles: 120 (5 min)) (Fig. 2b). As the result, for V_r_ = −0.8 and −0.4 V, H^+^ flow from the solution into the SPA and induce a reduction current of e^−^ to flow from the electrode. During such H^+^ doping into the SPA, the solution loses H^+^, resulting in a pH increase. For V_o_ = 0.8 and 0.4 V, we see an oxidation current that induces H^+^ injection into the solution and decreases the pH. In Fig. 2d, we summarize the net charge (${\sum }^{}It$, mC) from the oxidation and reduction currents. A V_r_ pulse applied to the SPA induces a negative net charge (−1.87 mC). Since protonic charge compensation occurs at the interface between SPA electrode and solution^32^, we assume this negative charge is wholly due to H^+^ doping, so an equal amount of H^+^ [18.8 nmol] from the solution transfers into the SPA, and from equation (1) the pH_surface_ changes from 6.5 to 6.92. We confirm the pH_surface_ change in a 10 mM Tris-HCl buffer solution by including 80 μM bromothymol blue as a pH indicator (Fig. 2e). The color of bromothymol blue changes from yellow at pH 6.5 to green at 7.0 (Fig. 2c). In Fig. 2e, the original yellow color at 0 min changes to green after 5 min, but only where adjacent to the SPA electrode. By contrast, applying a V_o_ pulse to the SPA drives a net charge of 1.54 mC and the calculated final pH returns to the original pH of 6.59. This is confirmed by the color change from green to yellow after 10 min. This reversible behavior can be observed during several repeated cycles. Thus, these results indicate that we can control pH on the SPA surface with an external voltage and also estimate a surface pH from the measured net H^+^ charge.

### pH control in mitochondrial matrix with pH modulation

**Figure 3:**
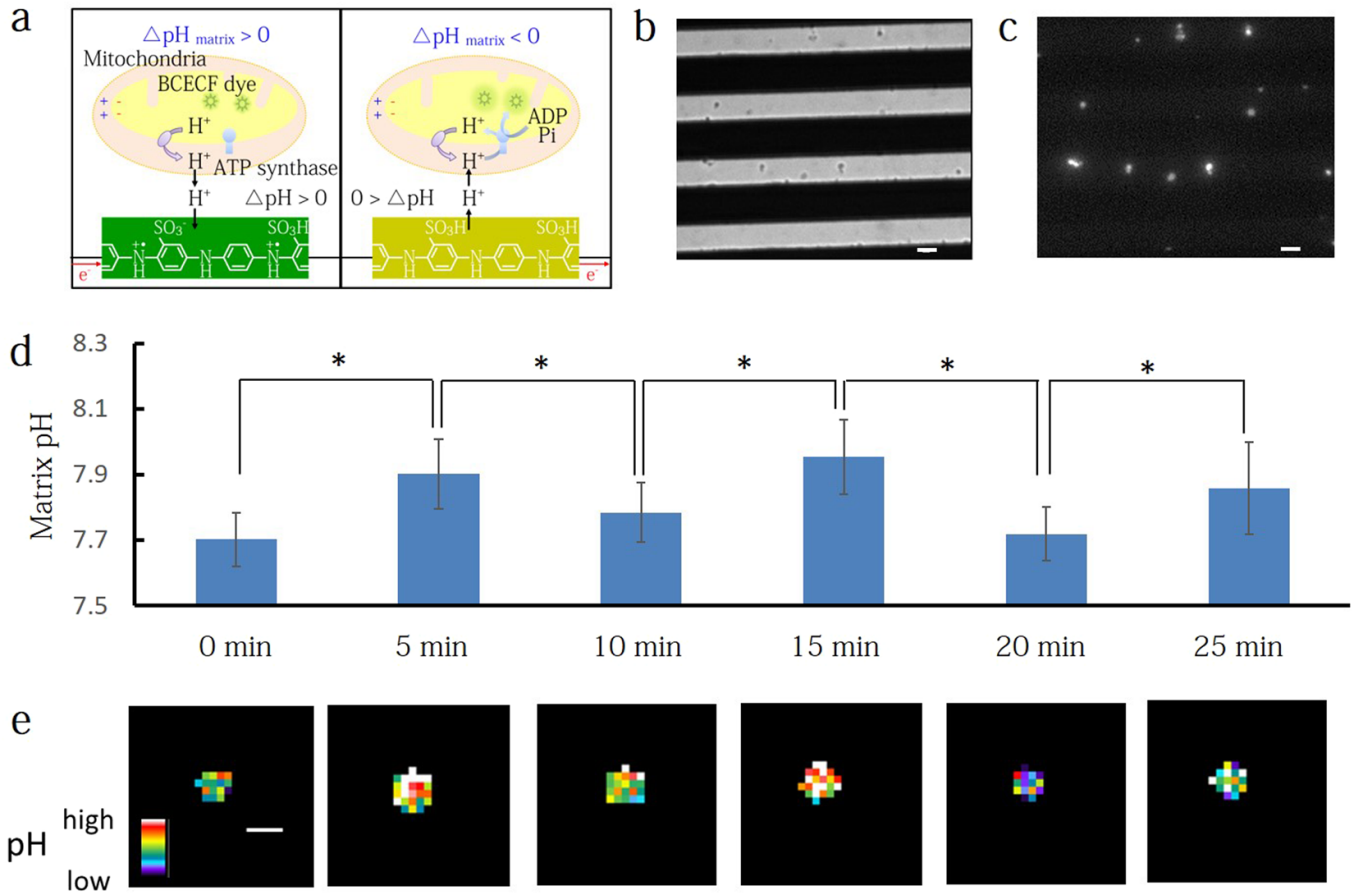
pH control in mitochondrial matrix with pH modulation. (**a**) Schematic of the pH modulator integrated with BCECF-labeled mitochondria. BCECF dye is a hydrophilic, dual-excitation ratiometric pH indicator that is trapped in the matrix and indicates the local pH through a pH-dependent ratio of emitted fluorescence at excitation wavelengths of 480 and 405 nm (F_480_/F_405_ ratio). (**b**,**c**) Transmittance and fluorescence images of the adsorbed mitochondria on the comb SPA microelectrode fabricated on a glass substrate. Black lines are where the SPA microelectrodes block the light transmission. White lines are the exposed glass substrate where adsorbed mitochondria can be observed. Scale bar: 5 μm. (**d**) The matrix pH fluctuated in response to doping and dedoping of H^+^ from SPA microelectrode. At t = 0, pH was 7.7. V_r_ pulse for H^+^ doping was applied for 5 min at t = 0, 10 and 20 min. V_o_ pulse was applied at t = 5, 15 and 25 min. The pH in 19 individual mitochondria was monitored with time. *P < 0.05. (**e**) Pseudo color images of the matrix pH estimated from the BCECF ratio at t = 0, 5 10, 15, 20 and 25 min. Scale bar: 2 μm.

To demonstrate that H^+^ transfer at the SPA transducer induces the pH modulation within the mitochondrial matrix (pH_matrix_), we integrated isolated mitochondria into our pH modulator (Fig. 3a). The mitochondria were isolated from pig heart using our previous procedures^37^. We physically adsorbed the mitochondria onto the glass substrate in the 7 μm gap between SPA comb microelectrodes at 4 °C for 2 h and then stained the mitochondrial matrix with 2,7-bis(2-carboxyethyl)-5,6-carboxyfluorescein (BCECF), a dual-excitation ratiometric pH indicator, in a pH 7.4 buffer containing 5 μM BCECF acetoxymethyl ester (BCECF-AM), 10 mM Tris-HCl, 250 mM sucrose and 0.5 mM ethylene glycol tetraacetic acid (EGTA). A BCECF-AM compound is a membrane permeant molecule. After cleaving the AM groups by esterase enzymes in a mitochondrial matrix, the BCECF indicator becomes trapped inside the matrix^38^. The trapped BCECF in the matrix indicates the local pH through a pH-dependent ratio of emitted fluorescence at excitation wavelengths of 480 and 405 nm (F_480_/F_405_ ratio). We measured the F_480_/F_405_ ratio in mitochondria at the differing solution pH (6.8, 7.3, 7.8, 8.3 and 8.8) in the presence of carbonyl cyanide m-chlorophenylhydrazone (CCCP) and then convert its ratio to pH value by the equation F_480_/F_405_ = (A + B × 10^(7-pH)^)/(C + 10^(7-pH)^) reported by Jameskracke^38^, where A is 3.12, B is 7.65 and C is 0.08 (see Fig. S3). The three parameters A, B and C depend on the instruments and the environment around the dye and are obtained by fitting the equation to the data shown in Fig. S3 with least-square method.

We optically observed that the mitochondria are adsorbed to the SPA comb microelectrode glass substrate, as shown by the transmittance (Fig. 3b) and fluorescence images (Fig. 3c). From these images, we confirm the location (proximity to the SPA microelectrode), shape, and quantity of mitochondria. After immobilization, we measure the value of F_480_/F_405_ from BCECF dye in the mitochondrial matrix while applying potentials V_r_ (−0.6 V for 2 s and 0.3 V for 0.5 s, cycles: 120 (5 min)) and V_o_ (0.6 V for 2 s and −0.3 V for 0.5 s, cycles: 120 (5 min)). For this measurement, we choose individual mitochondria located at the gap center, equidistant from the SPA comb microelectrodes. In Fig. 3d, at 0 min, the average F_480_/F_405_ ratio is 13.3, indicating a pH_matrix_ of 7.7. When V_r_ is applied, n(H^+^) = −10.95 nmol transfer from the solution to the SPA over a period of 5 minutes. After this time the average F_480_/F_405_ value increases to 16.9, indicating that the pH_matrix_ has increased to 7.9 due to H^+^ diffusion from the low pH mitochondrial intermembrane space to the high pH SPA surface. In addition, we confirmed this pH_matrix_ increase in individual mitochondria by estimating from the BCECF ratio (Fig. 3e). In contrast, when we switch the applied voltage to V_o_, n(H^+^) = 9.59 nmol injects into the solution and the average ratio and pH_matrix_ return to the original value of 13.3 and 7.7. This electrochemical pH modulation in the matrix can be cycled between the low pH (7.7) and the high pH (7.9) during several repeated cycles until the mitochondria lose their functions on the surface. During pH modulation, mitochondria maintained a significant membrane potential although a slight depolarization was observed through the decrease in tetramethylrhodamine ethyl ester (TMRE) fluorescence intensity (Fig. S4). These results indicate that we could electrochemically modulate pH in the mitochondrial matrix without the loss of mitochondrial membrane potentials.

### Control of mitochondrial ATP synthesis with pH modulation

**Figure 4:**
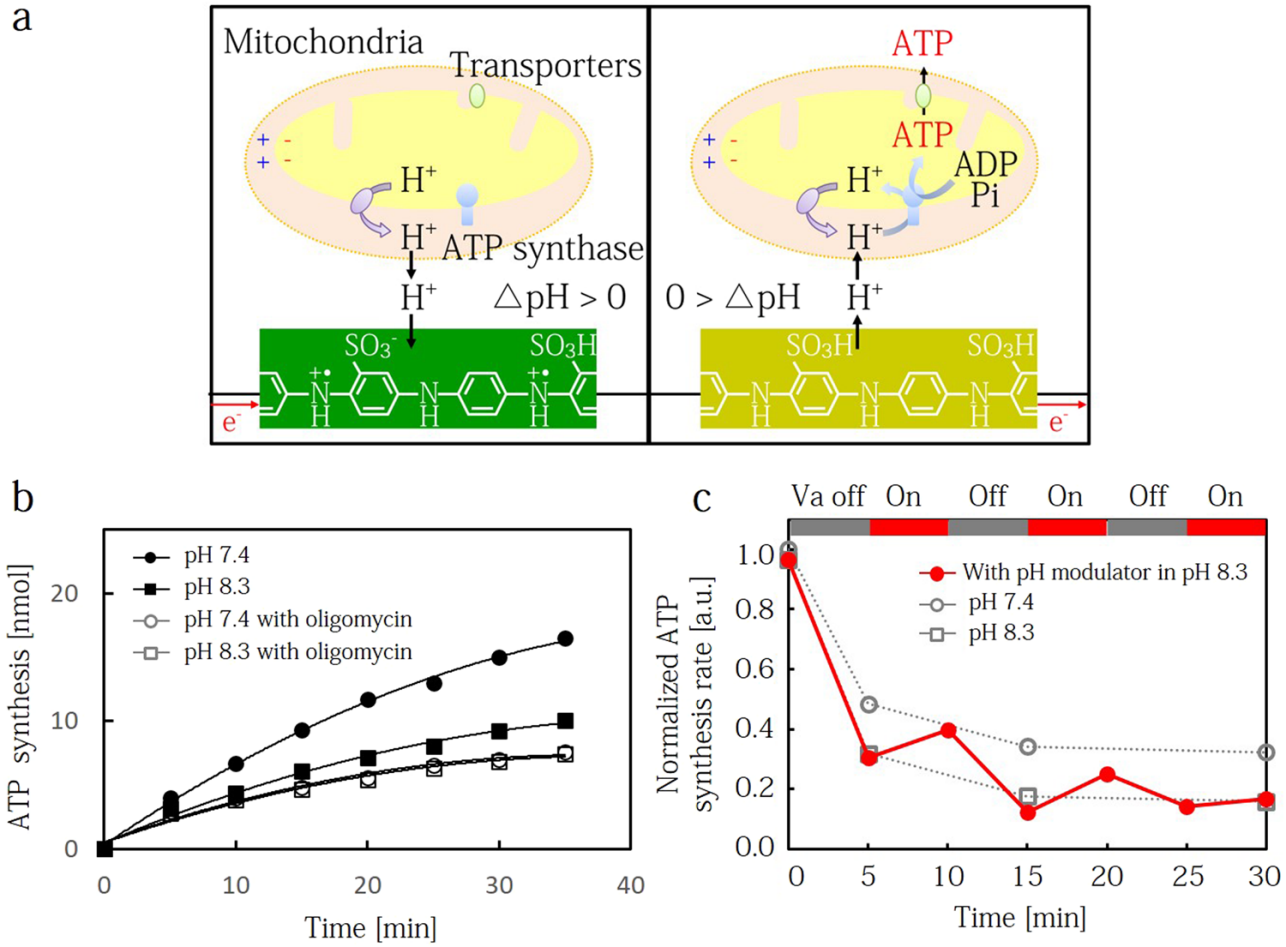
Control of ATP synthesis in mitochondria with pH modulation. (**a**) Schematic of the pH modulator integrated with the mitochondria. (**b**) ATP synthesis in mitochondria in pH 7.4 and pH 8.3 buffer solutions including 1 mM ADP and 1 mM P_i_. For comparison, the data includes experiments with oligomycin, which is an inhibitor of ATP synthase. The oligomycin results represent the baseline ATP production rate without ATP synthase. ATP product was expressed as luminescence intensity. (**c**) ATP synthesis rate in mitochondria is controlled by pH modulator in pH 8.3 buffer solution. At t = 0 min, 1 mM ADP and 1 mM P_i_ were injected into the solution. At t = 5, 15 and 25 min, V_r_ pulse for H^+^ injection was applied for 5 min to SPA electrode. The data include the synthesis rate in pH 7.4 and pH 8.3 buffer solutions. The rate of ATP synthesis at t = 0 min in pH 7.4 buffer solution is normalized to 1.

We demonstrated the control of ATP synthesis in mitochondria through pH modulation (Fig. 4). In the same manner as the matrix measurements, mitochondria adsorb on the SPA-coated Au electrode in 10 mM Tris-HCl buffer containing 250 mM sucrose and 0.5 mM EGTA. Before the measurement, we added a Tris-HCl buffer containing 1 mM ADP and 1 mM P_i_ to initiate ATP synthesis. We first measured the synthesized ATP molecules from mitochondria at varying solution pH by estimating the bioluminescent intensity from the firefly enzymatic reactions of ${\rm{luciferin}}+{\rm{ATP}}+{O}_{2}\,\to Oxyluciferin+AMP+P{P}_{i}+C{O}_{2}$ (Fig. 4b). After 35 min, the ATP product at pH 7.4 is 16.5 nmol, but only 10.0 nmol at pH 8.3. This is because the proton translocation across the inner membrane at pH 7.4 is more facilitated than that at pH 8.3. ATP synthesis in mitochondria is mainly produced by two enzymes: ATP synthase, which drives ADP oxidative phosphorylation $(\mathrm{ADP}+{P}_{i}\to \mathrm{ATP})$ and adenylate kinase, which drives ADP disproportionation ($2{\rm{ADP}}\to {\rm{ATP}}+{\rm{AMP}}$). To confirm that the observed ATP product is not the result of adenylate kinase activity at differing pH, we added 5 μM oligomycin into the Tris-HCl buffer solution as an inhibitor for ATP synthase (Fig. 4b, blank symbol). At either observed pH, the products (7.6 nmol at pH 7.4 and 7.4 nmol at pH 8.3 with olygomycin for 35 min) have similar values, and are much lower than the data without olygomycin. Thus, the difference in the ATP product for differing solution pH is solely due to the reaction from ATP synthase.

To perform the modulation of ATP synthesis in mitochondria with an SPA transducer, we measured the rate of ATP synthesis in mitochondria at the ON and OFF states of the applied potential V_o_ (Fig. 4c). We used pH 8.3 Tris-HCl buffer solution (10 mM) as the electrolyte, and the three electrode system using SPA WE, Ag/AgCl RE and Pt CE to control the solution pH on the SPA surface. We started the ATP measurement by injecting 1 mM ADP and 1 mM Pi substrates at t = 0, and evaluate the ATP product at 5 min intervals. The evaluated rate of ATP synthesis was normalized to 1 at t = 0 s. The initial rate of ATP synthesis decreases rapidly from 1.00 to 0.30 over 5 min because the activities of both ATP synthase and adenylate kinase are high due to the large initial concentration gradient of ADP substrate and ATP product at the mitochondrial intermembrane. After the initial 5 min, the rate of ATP synthesis decreases gradually over the next 30 min. The normalized rate in pH 8.3 Tris-buffer solution decreases from 0.30 to 0.16, while the normalized rate in pH 7.4 Tris-buffer solution decreases from 0.48 to 0.32. When directly controlled by applying V_o_ to the SPA, the rate of ATP synthesis is enhanced from 0.30 (at 5 min) to 0.39 (at 10 min) and from 0.12 (at 20 min) to 0.25 (at 25 min). If we do not supply any V_o_ to the SPA, we do not observe any noticeable modulation of the mitochondrial ATP synthesis rate. Since the activity of isolated mitochondria at room temperature decreases after 25 min, we could not modulate the mitochondrial activity beyond 30 min.

## Discussion and Conclusions

We have demonstrated a sulfonated polyaniline based protonic biotransducer that monitors and controls the pH dependent mitochondrial ATP synthesis. This transduction mechanism is based on the pH_surface_ dependence of the H^+^ transfer between the SPA polymer and solution. Since the SPA polymer has a high proton affinity, we can modulate the pH_surface_ reversibly from pH 6.5 to 7.0 with an external voltage applied to the SPA, and also evaluate the pH value in the buffer solution from the measured net H^+^ charge. Furthermore, we confirmed the difference between our SPA electrode and the common PdH_x_ protode by measuring the redox currents at the V_r_ (−0.5 and −0.9 V for 15 s) and the V_o_ = 0.3 V for 15 s in pH6.5 Tris-HCl buffer including 80 μM bromothymol blue as a pH indicator (Fig. S5). The main advantages of using a SPA protode are to obtain high H^+^ affinity due to functional sulfonic groups that avoid inducing side reactions and to enhance protonic currents due to a large surface area of three-dimensionally structured SPA deposited on comb microelectrodes.

The pH modulation induces H^+^ transfer between the mitochondria and SPA interface, changing the mitochondrial intermembrane proton concentration and therefore regulating the synthesis of ATP. This is the first demonstration of a H^+^ biotransducer connected to a living mitochondria and directly interfacing with the enzymatic activity. In future applications, our H^+^ biotransducer could integrate with pH dependent biological samples like ion channels^17^ and enzyme cascades^39^, and might allow further control of biological functions^40^.

## Materials and Methods

### Electrochemical polymerization of SPA polymer on Au comb microelectrodes

The microelectrodes are fabricated on glass slides. 100 nm Au with a 10 nm Cr adhesion layer is deposited via thermal evaporation, and comb micropatterning (7 μm width and 7 μm pitch) is carried out with photolithography. To polymerize SPA polymer on Au comb electrodes, we use a three electrode system (HOKUTO DENKO, HSV-110) with the comb electrodes as the working electrode, Ag/AgCl as the reference electrode and Pt as the control electrode. A cyclic potential is swept 10 times from −0.3 to 1.9 V (vs. Ag/AgCl) at 100 mV/s in a dehydrated acetonitrile solution that includes 0.2 M aniline solution and 0.2 M fluorosulfuric acid solution. After this polymerization step, the substrate is rinsed with distilled water, and then stored overnight in Tris-HCl buffer to maintain a target pH.

### Electrochemical pH modulation in solution

PDMS wells (made from 10 mL PDMS solution) are used as solution containers and attached to the SPA substrates with PDMS. To confirm pH color change, we add 0.5 mL of Tris-HCl buffer solution (pH 6.5) with 80 μM bromothymol blue to the PDMS well, then apply the periodic pulse voltages with a function generator (HOKUTO DENKO, HA-151B) and a potentiostat (HOKUTO DENKO, HB-305). V_o_ and V_r_ pulse are applied to the SPA, where V_o_ = 0.8 V for 2 s and −0.4 V for 0.5 s, Cycles: 120 (5 min), V_r_ = −0.8 V for 2 s and 0.4 V for 0.5 s, Cycles: 120 (total: 5 min). During the voltage supply, we record the applied voltage and the current at the WE with a data logger (GRAPHTEC, GL240).

### Mitochondria isolation from pig heart

Mitochondria are obtained from pig hearts freshly collected from a local slaughterhouse and isolated by differential centrifugation as previously described^37^. We store the mitochondria in a freezer and use it within one week. Each preparation is assayed for ATP production and confirmed by observing that ATP synthesis rate is comparable to previously reported values. Prior to the measurements, mitochondria are thawed and adsorbed onto the SPA comb microelectrode glass substrate. The adsorption of mitochondria are performed by incubation of mitochondria on microelectrode substrate for 2 h in the buffer (10 mM Tris-HCl, 250 mM sucrose, 0.5 mM EGTA, pH 7.4) and are washed twice before microscopic measurements. Adsorption and washing are performed at 4 °C. During 2 h incubation at 4 °C, ATP production activity of mitochondria are not decreased. Protein content is determined using a protein assay with BSA as a standard.

### Matrix pH control in mitochondria

The PDMS well is mounted on a SPA comb microelectrode substrate. Then, 10 ml isolated mitochondria in 10 mM Tris-HCl buffer solution containing 250 mM sucrose, 0.5 mM EGTA (pH 7.4) is added into PDMS well and adsorbed for 2 h at 4 °C. To indicate pH in the mitochondrial matrix, the adsorbed mitochondria is cultured in the buffer solution including 5 μM BCECF-AM for 15 min at 4 °C and washed with the buffer solution at 25 °C. The BCECF-labeled mitochondria is observed at 25 °C with a fluorescence microscope (OLYMPUS IX70) equipped with a 40× objective lens (Uapo 40×/340; NA = 0.90; Olympus Corporation) and a cooled CCD camera (Sensicam QE, PCO AG; Kelheim, Germany). BCECF is illuminated with a 75-w xenon lamp through a 30 nm bandpass filter centered at 405 nm or a 20 nm bandpass filter centered at 480 nm. Fluorescence from BCECF is collected between 515 and 550 nm. Total fluorescence of F_480_ and F_405_ from individual mitochondria are obtained as integrated fluorescence as described previously^41^. The fluorescence ratio (F_480_/F_405_) is measured in the presence of 5 µM carbonyl cyanide m-chlorophenyl hydrazine (CCCP) at the different solution pH (6.8, 7.3, 7.8, 8.3 and 8.8) to equilibrate the pH between buffer solution and mitochondrial matrix. To calibrate the pH from F_480_/F_405_ ratio, we fit the data with least-square fitting of F_480_/F_405_ = (A + B × 10^(7-pH)^)/(C + 10^(7-pH)^), where A is 3.12, B is 7.65 and C is 0.08. After the calibration, we apply the pulse voltages V_o_ and V_r_ to the SPA electrodes, where V_o_ = 0.6 V for 2 s and −0.3 V for 0.5 s, Cycles: 120, V_r_ = −0.6 V for 2 s and 0.3 V for 0.5 s, Cycles: 120. We measure the F_480_/F_405_ ratio during pH modulation.

### Control of mitochondrial ATP synthesis

10 ml isolated mitochondria is added to the PDMS well on the SPA substrate and adsorbed in 10 mM Tris-HCl buffer solution (pH 7.4 or pH 8.3) containing 250 mM sucrose, 0.5 mM EGTA for 2 h at 4 °C. Prior to the measurements, 1 mM Na malate and 1 mM Na glutamate are added to mitochondria and mitochondria were kept at 25 °C. At the beginning of the measurement (t = 0) we add 1 mM ADP and 1 mM P_i_ substrate for mitochondrial ATP synthesis into the PDMS chamber. At the same time, we collect a 0.1 ml sample from the solution in the mitochondria-adsorbed PDMS well and then determine the ATP concentration with a commercial kit (CellTiter-Glo Luminescent Cell Viability Kit, Promega, EI, USA) by measuring the bioluminescence with a luminescent meter (MLR-100 Micro lumino reader, Colona Electric, Ibaraki, Japan). Every five minutes we measure ATP concentration for each experimental setup: (a) with and without 5 μM oligomycin, (b) with the applied voltage V_r_ at 5, 15 and 25 min for 5 min, where V_r_ = −0.6 V for 2 s and 0.3 V for 0.5 s, Cycles: 120. The difference in pH of the buffer between 7.4 and 8.3 had no effects on the intensity of luminescence under the present condition.

## Electronic supplementary material

Supplementary materials
