# Supplementary material for: A protonic biotransducer controlling mitochondrial ATP synthesis
Source: Sci Rep. 2018 Jul 12;8:10423. doi: 10.1038/s41598-018-28435-5 (PMC6043558; doi:10.1038/s41598-018-28435-5)
Supplement: Supplementary file 1 — Supplementary materials [file 41598_2018_28435_MOESM1_ESM.pdf]

Supplementary Materials

A protonic biotransducer controlling mitochondrial ATP synthesis

Z. Zhang[1], H. Kashiwagi[2], S. Kimura[2], S. Kong[1], Y. Ohta[2*], T. Miyake[1*]

[1] Graduate School of Information, Production and Systems, Waseda University, Kitakyushu, Fukuoka, 808-0135, Japan

[2] Division of Biotechnology and Life Sciences, Institute of Engineering, Tokyo University of Agriculture and Technology, Koganei, Tokyo 184-8588, Japan

*Corresponding authors: miyake@waseda.jp and ohta@cc.tuat.ac.jp

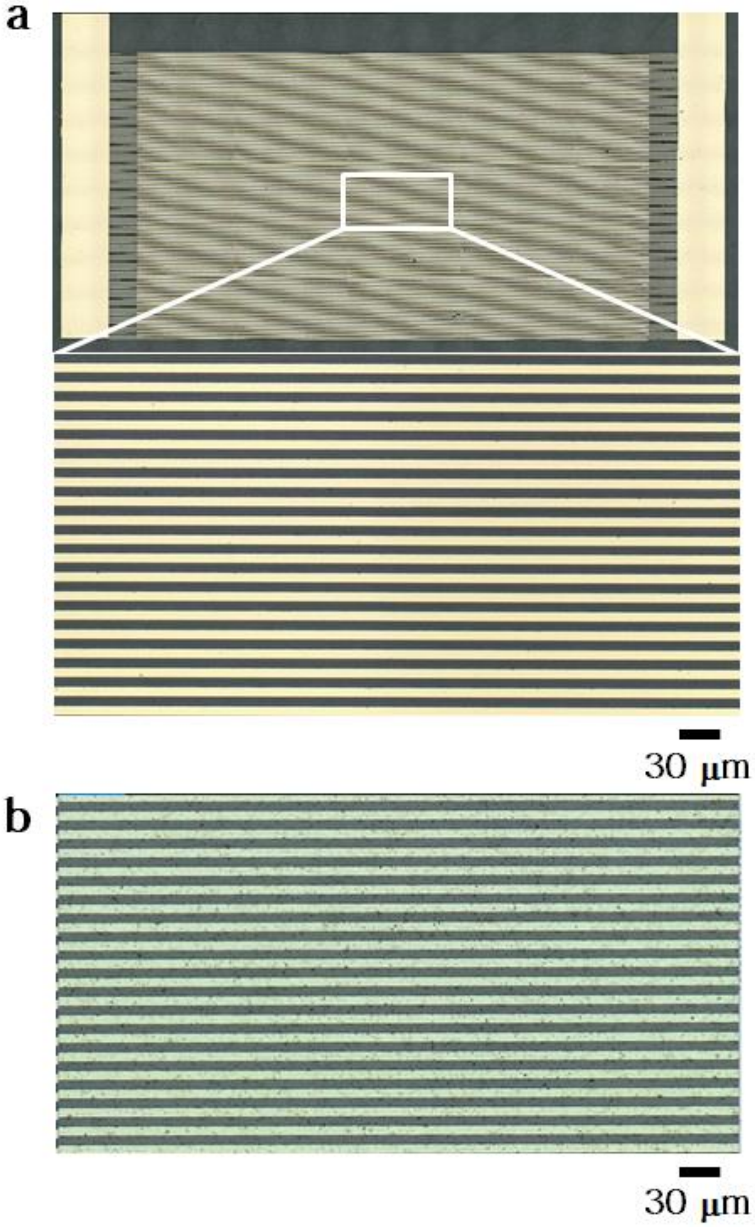

Figure S1. (a) Pictures of Au comb microelectrodes and (b) after SPA polymerization.

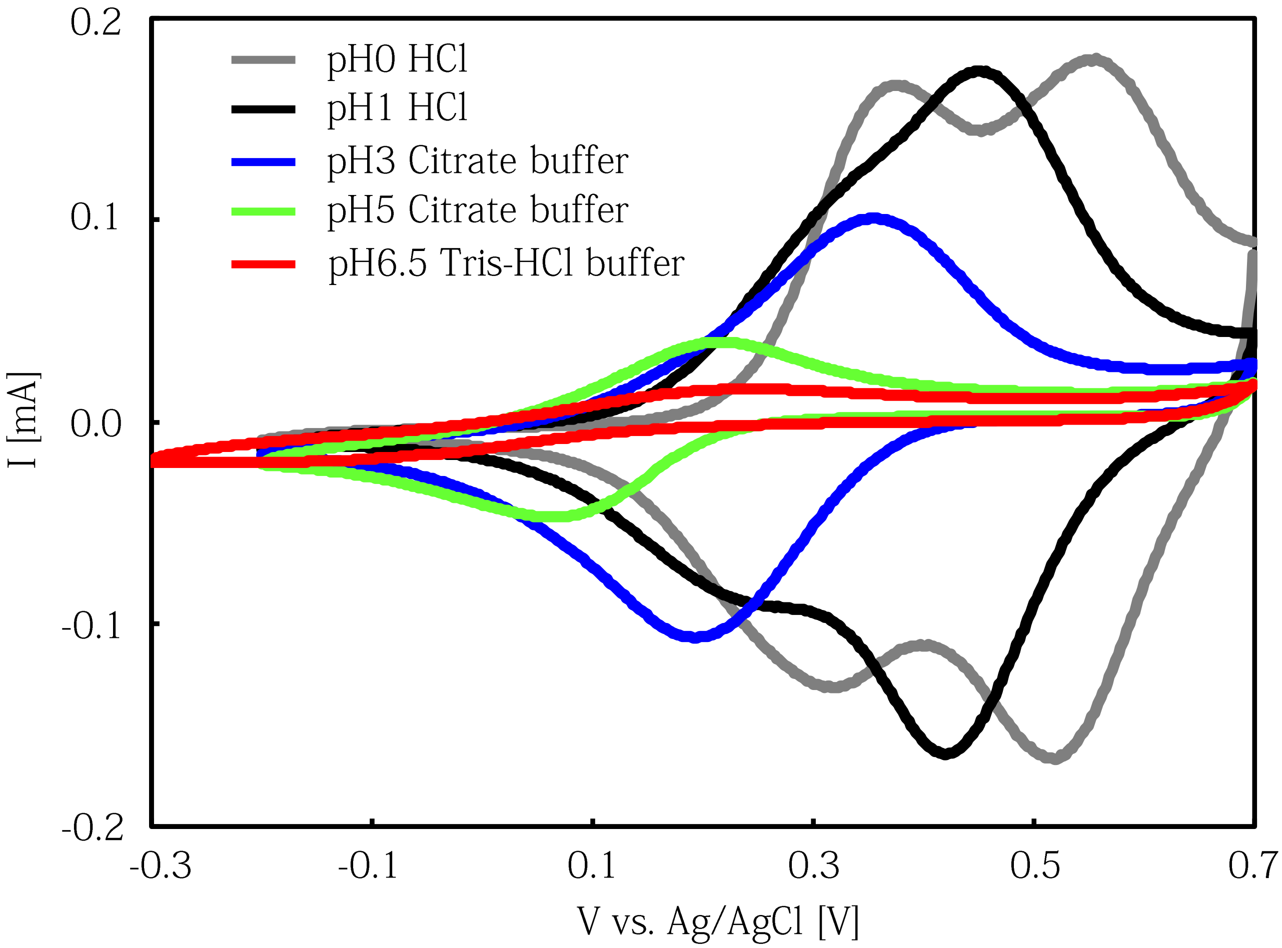

Figure S2. Cyclic voltammograms of the SPA microelectrode at 20 mV s^{-1} in the different pH solutions: pH0 HCl (gray line), pH1 HCl (Black), pH3 citrate buffer solution (Blue), pH5 citrate buffer solution (Green) and pH6.5 Tris-HCl buffer solution (Red).

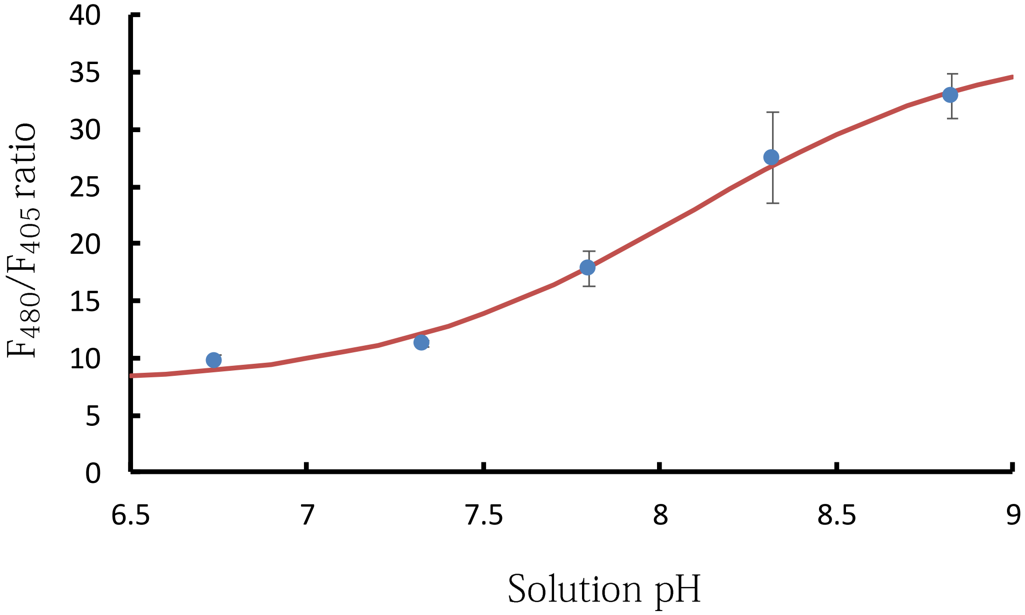

Figure S3. **Calibration curve for the determination of pH in the mitochondrial matrix.** Mitochondria stained with BCECF were incubated with 5 μM CCCP for 10 min in different pH solutions. Data are expressed as the mean ±S.D., N=3. The solid curve is the theoretical curve fitted with the least-squares method.

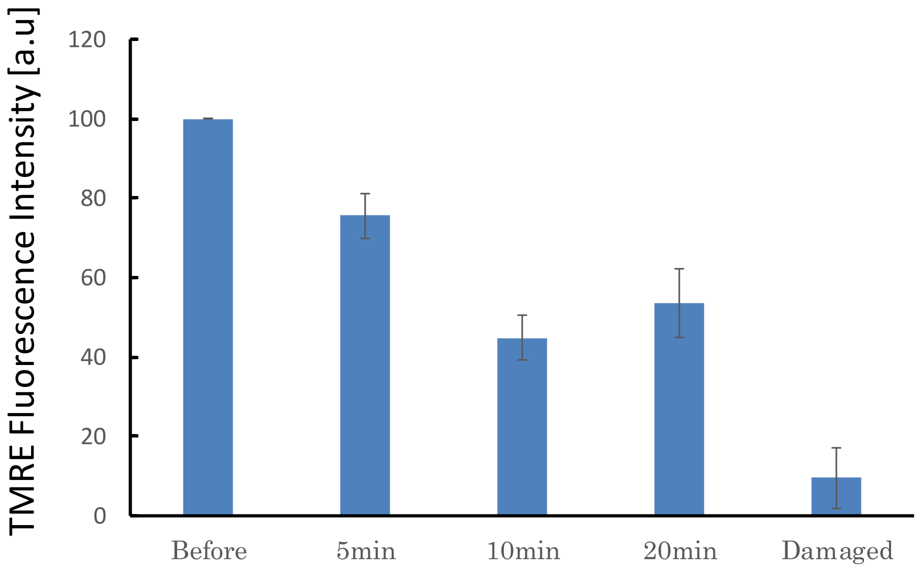

Figure S4. **Temporal TMRE fluorescence intensity during pH modulation indicates the mitochondrial membrane potential.** To observe mitochondrial membrane potential, mitochondria were stained with 20 nM TMRE, which percolate into the mitochondrial intramembrane and work as a membrane potential sensitive dye. TMRE fluorescence intensity in individual mitochondria were measured with fluorescence microscopy. The intensity at t = 0 s was normalized to 100. We measured the TMRE fluorescence intensity when we applied sequential voltages of V_c, V_a, V_c and 1.5 V_{damage}. The data were expressed as the mean ± S.D. N = 5.

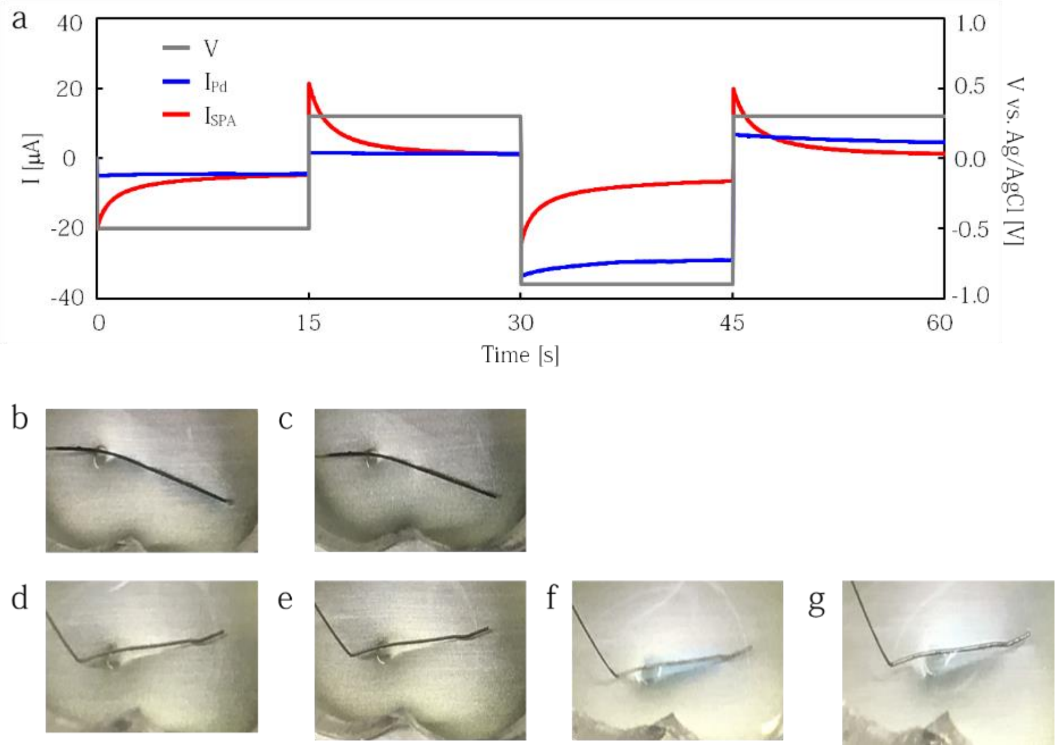

Figure S5. (a) Temporal redox currents of SPA-coated Au wire and Pd wire at sequentially applied voltages of -0.5, 0.3, -0.9 and 0.3 V. We used the same diameter (0.1 mm) and length (ca. 7 mm) for both wires. (b-g) Pictures of SPA-coated Au wire and Pd wire with pH dye in solution at t=15, 30, 45 and 60 s. (b) is an image of SPA-coated Au wire taken at t=15 with V_r = -0.5 V. A blue color is observed only in the vicinity of SPA surface. (c) When an oxidizing voltage V_o = 0.3 V is applied, the SPA adsorbs protons from the solution, so the blue color returns to the original yellow color. (d and e) are Pd images after applying the voltages of V_r = -0.5 V and V_o = 0.3 V, respectively. There are no color changes. (f) When a reducing voltage V_r = -0.9 V is applied to Pd wire, Pd adsorbs protons from the solution to form PdH_x. After that, we could observe the color change from yellow to blue color in the vicinity of the PdH_x surface. (g) When the voltage is returned to V_o = 0.3 V, the PdH_x injects protons into the solution, so the solution color changes to pale blue, but does not return to the original yellow color because of partial currents for side reactions including in the net currents during proton loading into Pd.
